# Prediction of COVID-19 with Computed Tomography Images using Hybrid Learning Techniques

**DOI:** 10.1155/2021/5522729

**Published:** 2021-04-22

**Authors:** Varalakshmi Perumal, Vasumathi Narayanan, Sakthi Jaya Sundar Rajasekar

**Affiliations:** ^1^Department of Computer Technology, MIT Campus, Anna University, Chennai, India; ^2^Melmaruvathur Adhiparasakthi Institute of Medical Sciences and Research, Melmaruvathur, Chengalpattu District, India

## Abstract

Reverse Transcription Polymerase Chain Reaction (RT-PCR) used for diagnosing COVID-19 has been found to give low detection rate during early stages of infection. Radiological analysis of CT images has given higher prediction rate when compared to RT-PCR technique. In this paper, hybrid learning models are used to classify COVID-19 CT images, Community-Acquired Pneumonia (CAP) CT images, and normal CT images with high specificity and sensitivity. The proposed system in this paper has been compared with various machine learning classifiers and other deep learning classifiers for better data analysis. The outcome of this study is also compared with other studies which were carried out recently on COVID-19 classification for further analysis. The proposed model has been found to outperform with an accuracy of 96.69%, sensitivity of 96%, and specificity of 98%.

## 1. Introduction

**Figure 1:**
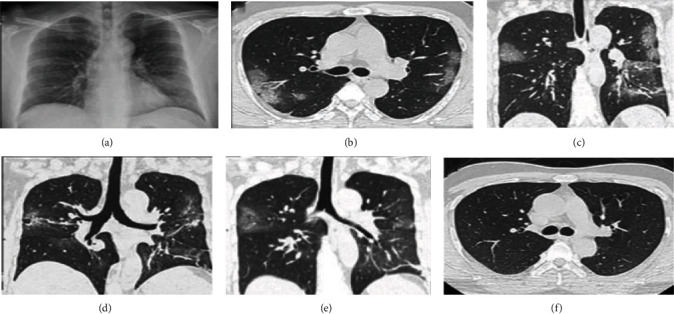
CT scan images of a COVID-19 patient as time goes by. (a) 7th Day (as soon as symptoms show up): CT scan presents opacities formed in left lower lobe and right upper lobe. (b, c) 9th Day: CT scan depicts ground-glass opacities which are bilateral and multifocal. (d) 15th Day: CT scan presents that virus has evolved into mixture of consolidations and opacities. (e) 19th Day: CT scan shows partial disappearance of ground-glass opacities and consolidations using antiviral treatments. (f) 31st Day: CT scan shows absence of pleural effusions, pulmonary cavitation, and lymphadenopathy [2–4].

The COVID-19 virus, believed to have initially originated from the Phinolophus bat, transmitted to human beings in December 2019. Wuhan city's Huanan Seafood Market was the nerve center for the COVID-19 outbreak which spread rapidly all around the world [1] and was eventually announced as a pandemic by World Health Organization (WHO) during March 2020 [2]. COVID-19-infected individuals have experienced severe acute respiratory disorders, fever, continuous coughing, and other infections. The mortality rate of this pandemic reached its peak in a short span of time. Early detection of the COVID-19 virus is the best way in mortality reduction. The CT scan images of COVID-19-affected individuals show distinctive characteristics like patchy multifocal consolidation, ground-glass opacities, interlobular cavitation, lobular septum thickening, and clear indication of fibrotic lesions, peribronchovascular, pleural effusion, and thoracic lymphadenopathy. The evolution of consolidation and ground-glass opacities over a period of time of a COVID-19-affected patient from symptom commencement to the next 31 days is delineated in Figure 1 [2–4]. RT-PCR is known to be the standard testing tool but has produced false negative rates in recent studies [5, 6] at the early stages. Studies also postulated the importance of CT scan images to screen COVID-19 with better specificity and sensitivity [7].

The characteristics of COVID-19 are similar to other viral pneumonia [4]. Yet with help of deep learning techniques, one can predict the differences between types of viral pneumonia precisely. The main differences between pneumonia caused by different types of viruses including the Respiratory Syncytial Virus (RSV) and Human Metapneumovirus (HMPV) in terms of ground-glass opacity (GGO), consolidation, and pleural effusion are depicted in Table 1. +++ is 50% area of lungs being involved and + is 10% area of lungs being involved.

The large number of CT scan images opens up a research area for start-up companies. These techniques proposed by researchers aid radiologists and physicians for fast and early prediction of the disease.

RT-PCR which is used for diagnosing COVID-19 has a few limitations. Firstly, the test kits are not sufficiently available and consume more time for testing, and the sensitivity of testing varies. Thus, using CT scan images for screening COVID-19 is important. CT scans images expose patchy ground-glass opacities which are hazy white spots in the lungs, which is the primary sign of COVID-19. In a recent study [8], with 1,014 patients, deep learning technique was able to predict (888/1014) positive cases using CT scan images of suspected COVID-19 patients, while RT-PCR was only able to predict (601/1014) positive cases of suspected COVID-19 patients. The results have shown that the CT scan images were able to diagnose COVID-19 effectively thus saving more lives. The mortality rates for different CoV viruses are discussed in Table 2. There is little knowledge on what will be the future of the outbreak. There are different manifestations of COVID-19 as discussed in a study [9]. In a study [10], it was found that CT scans had a high sensitivity while diagnosing for COVID-19. CT scan of the chest is considered to be an important tool for COVID-19 detection in endemic regions. As a result of the sensitivity and specificity of CT scans, a clinical detection threshold based upon ideal CT scan imaging manifestations is now utilized in China. So, CT scan images act as a better alternative to RT-PCR testing. Thus, chest CT scan images can be utilized as a primary resource for detecting COVID-19 in endemic regions which lack access to the testing kits.

This also takes less time thereby saving radiologist's time for carrying out the further treatments. The following conclusions were arrived from the researchers carried out by many studies mentioned above:

1. The sensitivity and specificity of chest CT scans to screen COVID-19 is high. Thus, in endemic regions one can use the automated system that detects COVID-19 precisely
2. Chest CT scan images play a vital role in monitoring and evaluating COVID-19 patients with extreme and severe respiratory symptoms. Based on CT scans, the intensity of the lung infection and the time taken by the disease to evolve were assessed and discussions on treatments were made accordingly
3. Patients infected with COVID-19 require multiple chest CT scan images during the treatment to find the progression of the disease. Analysing multiple CT images is a time-consuming task and it cannot be completed with greater precision manually. Thus, screening many images quickly is a priority which is achieved through deep learning techniques
4. The prime abnormalities which are developed after the onset of symptoms in COVID-19-affected patients are ground-glass opacities (GGO), consolidations, and nodules. These features are easily recognizable through deep learning techniques
5. Early detection of COVID-19 infection is critical for treatment mitigation and safety control. When compared with RT-PCR, testing with chest CT images are more dependable, rapid, and practical methodology to scan and monitor COVID-19 patients, specifically in the hotspot regions
6. Even when the symptoms are not visible (asymptomatic), CT findings can detect visible changes and series of abnormalities in COVID-19-affected lungs using proposed model

So far, medical and clinical studies on chest CT scan findings have been discussed. In Table 3, the deep learning techniques which were carried out using images are presented.

The accuracy of the works is also shown along with the classification methods that were used. The predominant works delineated in Table 3 show 94.52% accuracy when model was built for CT images. It is also seen that most of the models are built for X-ray images [17–26]. Studies have shown instances where patient's chest X-ray showed no traces of lung nodules but then were later identified using CT scans [13, 15]. CT images play a major role in detecting the COVID-19 infection. Hence, for the above reasons, a hybrid learning model was proposed which scans the CT images and classifies them as COVID-19, CAP, and Normal images using machine learning and deep learning techniques.

## 2. Materials and Methods

**Figure 2:**
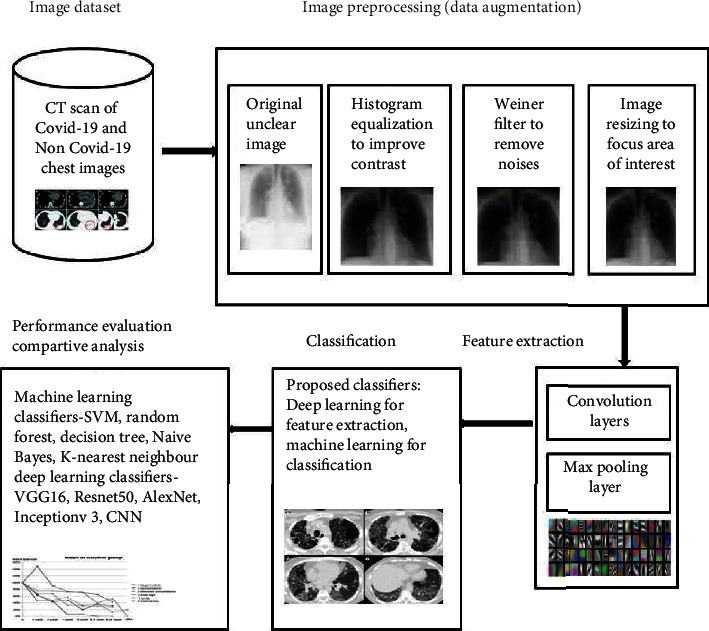
System architecture that was proposed for screening COVID-19 chest CT scans using feature extraction and classification.

Figure 2 shows the overall progression of the proposed hybrid learning model. The CT scan input images are collected from various sources like Google Images, RSNA, and Github, so they are different in resolution, size, and many other features. So, all the CT scan input images are preprocessed to standardize the images and given to the pretrained deep learning models for feature extraction. The extracted features are then given to machine learning classification models. The pretrained deep learning models used in the proposed work are VGG-16, Resnet50, InceptionV3, and AlexNet. The machine learning models used in the proposed work are Support Vector Machine (SVM), Random Forest, Decision Tree, Naive Bayes, and *K*-Nearest Neighbour (KNN).

### 2.1. Image Processing

**Figure 3:**
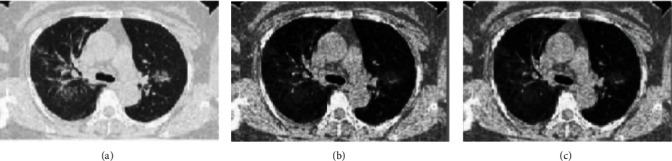
(a) Original COVID-19 CT scan image (b) Histogram equalized COVID-19 CT scan image (c) Weiner filtered COVID-19 CT scan image.

Figure 3 shows the progression of image processing.

**Figure 4:**
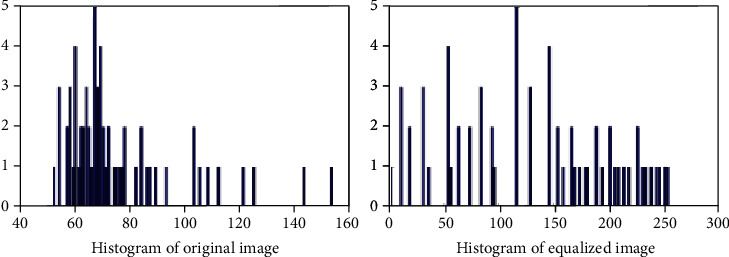
Histogram of original and equalized images.

The histogram equalization is applied to enhance the quality of the image without losing the important features of the image. The histograms of the original and equalized image are shown in Figure 4. The Weiner filter is used to remove the noises from the image yet preserving fine details and edges of the lungs. The filter size is chosen to be 4 × 4 in order to prevent the image from getting over smooth. Weiner filter is typically based on estimation of variance and mean from the local adjacent of individual pixels. It then constructs pixel-based linear filters using the Eq (1).

(1) $$\begin{array}{c} \text{WF}\left(i,j\right)=\mu +\frac{\sigma^{2}-v^{2}}{\sigma^{2}}.\left(O\left(i,j\right)-\mu \right), \end{array}$$

where WF(*i*, *j*) denotes the position of pixel in filtered image and *O*(*i*, *j*) denotes the position of pixel in the original image. *μ* and *σ* are mean and variance of local adjacent pixels, respectively. *v* is called the noise variance. Images are then resized to focus on a specific area of interest in order to extract its features.

### 2.2. Feature Extraction

**Figure 5:**
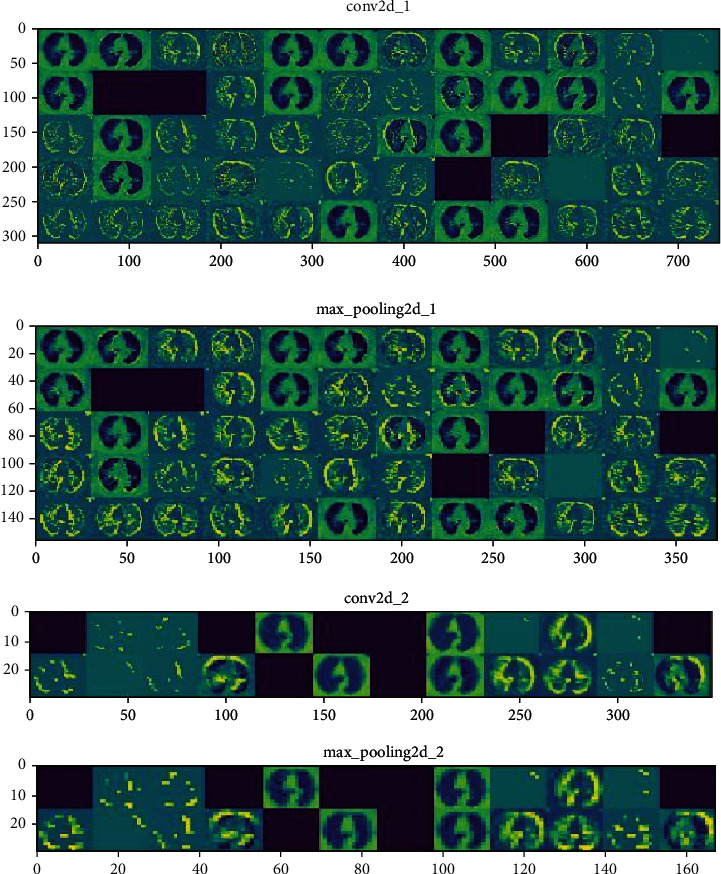
Convolutional and max-pooling layer intermediate image.

Feature extraction is achieved using pretrained CNN models such as VGG-16, Restnet50, InceptionV3, and AlexNet. CNN models are purposely used for image classification. An image is viewed as an array of pixel which also depends upon the resolution of an image. These CNN models consist series of convolutional and pooling layers. The data augmentation is achieved using a convolutional layer. The convolution operation is applied to a region of an image, sampling the values of the pixels in that particular region and converting them into a solitary value. This convolution operation is defined in Eq (2) and Figure 5.

(2) $$\begin{array}{c} E\left(i,j\right)=\overset{K}{\underset{a=-K}{\sum }}\overset{K}{\underset{b=-K}{\sum }}I\left(a,b\right)F\left(i-a,j-b\right), \end{array}$$

where *E*(*j*, *j*) is the value of pixel at (*i*, *j*) after convolution operation; *I*(*a*, *b*) is the value of pixel at (*a*, *b*) in input matrix and *F*(*i* − *a*, *j* − *b*) is the value of pixel at (*i* − *a*, *j* − *b*) in filter (Kernel) matrix and *K* is the kernel size or size of the filter matrix.

The output size of the convolution layer is given in Eq (3).

(3) $$\begin{array}{c} M=\frac{I-F+2P}{S}+1, \end{array}$$

where *M* is the size of the output matrix, *I* is the size of the input matrix, *F* is the size of the convolution filter, *P* is padding, and *S* is stride value for convolution operation. The max-pooling layer performs dimensionality reduction. This layer will downsample the value without losing any important information. It does max operation by finding the maximum valued neuron in a particular region for the output from the previous layer which is given in Eq (4) and Figure 5.

(4) $$\begin{array}{c} P\left(i,j\right)=\overset{a=M}{\underset{a=-M}{\sum }}\overset{b=M}{\underset{b=-M}{\sum }}\max\left(E\left(a,b\right)\right), \end{array}$$

where *P*(*i*, *j*) is the value of pixel at (*i*, *j*) after pooling operation is performed; *E*(*a*, *b*) is the value of pixel at (*a*, *b*) of preceding layer's output and *M* is the size of previous layer's output grid. The output size of the max-pooling layer is given in Eq (5).

(5) $$\begin{array}{c} N=\frac{M-F}{S}+1, \end{array}$$

where *N* is the size of the output matrix, *M* is the size of the previous layer's matrix, *F* is the size of the pooling filter, and *S* is the stride value same as what was chosen for convolution operation. Relu acts as an activation for convolutional and max-pooling layer as given in Eq (6)

(6) $$\begin{array}{c} F\left(x\right)=\max\left(0,x\right), \end{array}$$

where *x* is the input value provided to activate the neuron. Thus, all the parameters which were extracted from the series of convolution and pooling operations from all the pretrained models that were used for feature extraction only are shown in Table 4. One can notice that nontrainable parameters are less and only trainable parameters are used by the backpropagation algorithm to optimize and update the values of weight and bias. Thus, only the important features are utilized for training the model. The features are nonredundant and informative values are intended to facilitate precise diagnosis of classes.

### 2.3. Classification

Classification refers to a predictive modelling problem where a class label is predicted for an input image. The classification is performed using traditional machine learning classifiers by removing the fully connected layers from the pretrained deep learning models. The extracted features were utilized for the final classification using Support Vector Machine (SVM), Decision Tree, Naive Bayes, *K*-Nearest Neighbour (KNN), and Random Forest. In SVM, the input values are plotted in an *n*-dimensional space, and the optimal hyperplane that differentiates the classes is found. In Random Forest, a large number of decision trees are built to operate as an ensemble model where all decision trees predict the class label and eventually the class that gets more votes will be chosen as the predicted label. In Decision Tree, each node acts as a splitting criterion and the branches lead to the final node (leaf node) to provide the output. Naive Bayes is a conditional probability model which used the Bayes theorem for classification. KNN is a nonparametric classifier which classifies images based on its *k*-nearest neighbours.

## 3. Results and Discussion

In this section, datasets that have been utilized for carrying out the experiments are discussed. Further, the comparative analysis of results is discussed.

### 3.1. Data Formulation

The dataset used here contains CT scan images for COVID-19 (includes both symptomatic and asymptomatic), CAP, and normal chest CT scan images. The images were assimilated from multiple resources for training the model precisely. The data collected from different resources are shown in Table 5. Scanning scheme used for scanning the image is diverse thus the model is able to learn all possible images. Image preprocessing has been applied to make the dataset a standardized one. A total of approximately 500 CT scan images were obtained for each class to maintain the data balance. The images were split for training, validation, and testing purposes which are shown in Table 6. The project was conducted on windows platform using the Python software (Python Jupyter Notebook). Different packages like pandas for data loading and data accessing, numpy for array (matrix) creation, scikit-learn for machine learning classifiers, Keras's Tensorflow for deep learning classifiers, and matplotlib for plotting graphs are used in the implementation of the proposed work. These tools have been helpful in completely satisfying the requirements producing promising results.

### 3.2. Experimental Results

The COVID-19 images are correctly classified by the present model with greater precision and recall. Initially, about 111 images were tested for machine learning models like Support Vector Machine (SVM), Decision Tree, Naive Bayes, *K*-Nearest Neighbour (KNN), and Random Forest. Secondly, the images were trained and tested for deep learning models such as CNN, AlexNet, VGG-16, InceptionV3, and Resnet50. On further analysis, the fully connected layers for CNN models were removed, and the prediction was performed with machine learning models as hybrid learning models. This showed that the hybrid learning models such as AlexNet+SVM and AlexNet+Random Forest models yielded better results when compared with other models.

**Figure 6:**
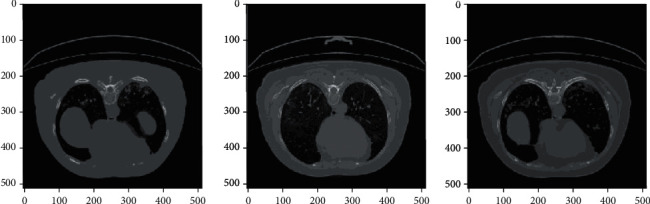
Colormap for COVID-19-affected chest CT scan images correctly classified by AlexNet+SVM and AlexNet+Random Forest.

**Figure 7:**
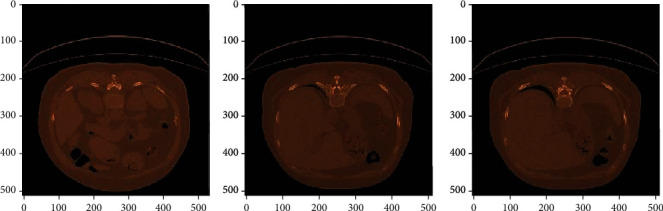
Colormap for CAP-affected chest CT scan images correctly classified by AlexNet+SVM and AlexNet+Random Forest.

**Figure 8:**
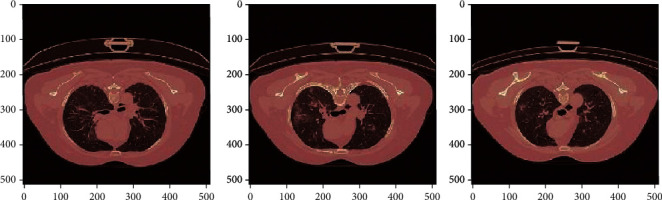
Colormap for normal chest CT scan images correctly classified by AlexNet+SVM and AlexNet+Random Forest.

Figure 6 shows the colormap images for COVID-19-affected CT scan images which were correctly classified by AlexNet+SVM and AlexNet+Random Forest.  Figure 7 shows the correctly classified CAP images, and Figure 8 shows the correctly classified normal CT scan images. These images in Figures 6–8 show the infected region in CT scan images which are then classified as CAP or COVID-19. The normal CT scan image does not have any infected region pointed in the image. The COVID-19 image shows an infected region in the left lower lobe region. This identification of the infected region is performed using Jet Colormap and Turbo heat map provided in python.

**Figure 9:**
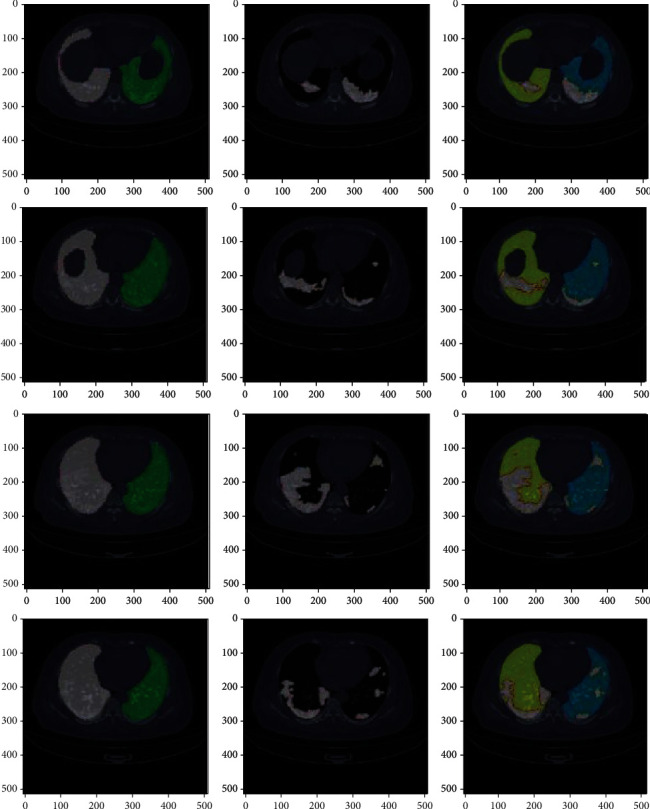
Images that were tested as negative by RT-PCR were actually positive cases and were correctly predicted as positive by the proposed work.

To compare this work with RT-PCR, 12 sample images of 3 patients are taken to test the model. All these images in Figure 9 are classified correctly by AlexNet+SVM and AlexNet+Random Forest, which are found to be negative by RT-PCR. The infected regions are also shown in the images using the colormap function provided by python.

Various metrics used to analyse different models are discussed below. F1-score, precision, and recall are defined in Eq (7), Eq (8), and Eq (9). Accuracy of a model shows how correctly the images are classified. The precision of the model determines the reproducibility of values or how many values are predicted correctly. Recall of a model shows how many correct values are discovered among all classes. F1-score takes precision and recall into account in order to calculate a balanced average value.

(7) $$\begin{array}{c} F1-\text{score}=2.\frac{\left(\text{precision}*\text{recall}\right)}{\left(\text{precision}+\text{recall}\right)}, \end{array}$$

where precision and recall are defined in Eq (8) and Eq (9). These values are in fact calculated from a Confusion matrix that is built using test data images.

(8) $$\begin{array}{c} \text{precision}=\frac{T_{p}}{T_{p}+F_{p}} \end{array}$$

where *T*_*p*_ is the number of images observed as positive and predicted as positive and *F*_*p*_ is the number of images observed as negative and predicted as positive.

(9) $$\begin{array}{c} \text{recall}=\frac{T_{p}}{T_{p}+F_{n}}, \end{array}$$

where *T*_*p*_ is the number of images observed as positive and predicted as positive and *F*_*n*_ is the number of images observed as positive and predicted as negative. Recall is also called sensitivity.

The specificity is defined in Eq (10).

(10) $$\begin{array}{c} \text{Specificity}=\frac{T_{n}}{T_{n}+F_{p}}, \end{array}$$

where *T*_*n*_ is the number of images observed as negative and predicted as negative and *F*_*p*_ is the number of images observed as negative and predicted as positive.

The accuracy for all the models can be calculated by Eq (11).

(11) $$\begin{array}{c} \text{Accuracy}=\frac{T_{p}+T_{n}}{T_{p}+T_{n}+F_{p}+F_{n}}, \end{array}$$

where *T*_*n*_ is the number of images observed as negative and predicted as negative. The Root Mean Square Error (RMSE) value for all the images can be evaluated using Eq (12).

(12) $$\begin{array}{c} \text{RMSE}=\sqrt{\frac{\sum_{j=1}^{n}{\left(y_{j}-y_{j}^{\prime }\right)}^{2}}{n}}, \end{array}$$

where *y*_*j*_ is the actual value, *y*′_*j*_ is the predicted value, and *n* is the total number of images.

The Mean Absolute Error (MAE) can be calculated using [26].

(13) $$\begin{array}{c} \text{MAE}=\frac{\sum_{j=1}^{n}\left|y_{j}-y_{j}^{\prime }\right|\;}{n}, \end{array}$$

where *y*_*j*_ is actual value, *y*′_*j*_ is predicted value, and *n* is the total number of images.

The Confusion matrix is often used to analyse the performance of the classification models using predicted class label for test images against known class label for test images. Classification report is used to evaluate the quality of prediction of class labels by classification models. The Confusion matrix and classification report for the models built using conventional machine learning classifiers are presented in Table 7. It is obvious that Random Forest has produced better results with the precision of 0.95, recall of 0.96, and specificity of 0.97 when compared with other machine learning classifiers. The Confusion matrix and classification report for models constructed using deep learning techniques are analysed and shown in Table 8. It is seen that AlexNet has produced better prediction outcomes with the precision of 0.94, recall of 0.94, and specificity of 0.97. The Confusion matrix and classification report for the proposed hybrid learning models are presented in Tables 9–13. The proposed works performed better than other classifiers. AlexNet+SVM has produced better results with the precision of 0.96, recall of 0.96, and specificity of 0.98 when tested for 333 test images. Resnet50+Random Forest has also produced better outcomes with precision, recall, and specificity of 0.95, 0.95, and 0.97, respectively. The feature extraction produces only necessary features to be trained and remove unnecessary features that are not vital for the classification task. This also helps the model to be faster in training and testing the classification models.

The outcomes of the models when trained for images before preprocessing and after preprocessing are also compared. There is a visible difference in results and it shows the significance of preprocessing the images. This analysis report is presented in Table 14. When comparing the outcome with studies that are featured in Table 3, the presented hybrid learning models have produced better results.

Thus, the prediction of COVID-19 using the classification model has been constructed in a robust way and it helps in quicker prediction of COVID-19. AlexNet model takes 13 minutes 25 seconds for training and 6 minutes 38 seconds for testing. VGG-16 model takes 20 minutes 43 seconds for training and 12 minutes 30 seconds for testing. InceptionV3 model takes 34 minutes 12 seconds for training and 20 minutes 12 seconds for testing. Resnet50 model takes 43 minutes for training and 21 minutes for testing. As the model gets deeper, it takes more time to train and test the images. The time taken to run the models is inversely proportional to a number of layers. RT-PCR which is used as a standard reference takes 1-2 days in India for confirming a patient to be infected by COVID-19 or not. When compared to RT-PCR, the present model aids in quicker prediction and can aid radiologist in carrying out further treatment and procedures. The accuracy of this model is also quite promising to perform the prediction when compared with RT-PCR. The CT scan images that were tested as negative by RT-PCR are also correctly predicted by these models. Often, the medical images are unclear with lesions and tissues being captured in CT scan images which can impede the prediction task. In order to overcome these difficulties, various image preprocessing techniques were applied. The image preprocessing techniques that are incorporated has an impact on accuracies and results. These techniques provide better resolution, high quality, and high-definition images for carrying out the prediction. In conclusion, the models presented in this study have produced better results in terms of outcomes (accuracy) and quicker prediction even in the early stages. In short, the proposed work can be used for this global public health emergency situation which requires immediate attention.

## 4. Conclusion

Early detection of COVID-19 is vital for treating and isolating the patients in order to avoid the spread of the virus. RT-PCR is contemplated as the standard technique, but it is reported that chest CT could be used as a rapid and reliable approach for scanning of COVID-19. The proposed hybrid learning models are able to detect COVID-19 with chest CT scan images with an accuracy of 96.69%, sensitivity of 96%, and specificity of 98% for AlexNet+SVM model. Even though there is overlap in patterns of abnormalities in CAP- and COVID-19-affected CT scans, these models are capable of performing well with greater accuracy, sensitivity, and specificity using multisource data assimilation. Finally, reliable models are proposed to distinguish COVID-19 and CAP from CT scan images.

## Figures and Tables

**Table 1:** CT findings for major types of viral pneumonia.

Infections	Transmission	GGO	Consolidation	Nodule	Pleural effusion
Adenovirus	Respiratory, oral-fecal	+++	+++	Centro lobular	C
Influenza	Droplet, airborne	+	+	++	UC
RSV	Aerosol, contact	+	+	Centrilobular +++	C
SARS-CoV2	Airborne, contact	+	+++	Rare	Rare
HMPV	Contact, droplet	+	+	Centrilobular +++	UC

**Table 2:** Severe Acute Respiratory Syndrome (SARS) versus Middle East Respiratory Syndrome (MERS) versus COVID-19.

CoV	Year	Origin	Mortality rate	Community attack rate	Incubation time
SARS	2002	Saudi Arabia	10%	30%-40%	4-14 days
MERS	2013	Saudi Arabia	34%	10-60%	7 days
COVID-19	2019	China	3.4%	4-13%	6 days

**Table 3:** Comparison of studies on COVID-19 classification.

Author	Image	Accuracy	Classification
Wang [11]	CT	82.9%	Transfer learning
Zhao [12]	CT	85%	DenseNet
Vruddhi Shah [13]	CT	94.52%	VGG-19
He X [14]	CT	94%	Self-trans model
Michael J. Horry [15]	CT	84%	Fine-tuned VGG-19
Song Ying [16]	CT	93%	Deep CNN

**Table 4:** Model parameter comparison.

Model	Image size	Total parameters	Trainable parameters	Nontrainable parameters	Number of layers
VGG-16	14,882,883	1,66,403	14,716,480	16	224 × 224
InceptionV3	22,370,339	5,62,691	21,807,6487	48	299 × 299
Resnet50	24,155,267	5,62,691	23,592,576	50	224 × 224
AlexNet	62,378,344	2,29,123	62,149,221	8	227 × 227

**Table 5:** Multisource data assimilation for COVID-19, CAP, and normal CT scan images.

Categories	Source	Images
COVID-19	medRXiv, bioRxiv, NEJM, JAMA, Lancet Medical Segmentation	349
	Coronacases	100
	Radiopaedia	10
	Zenodo	9
Total		488
CAP	Google Images, RSNA	500
Normal	Google Images, Github	500

**Table 6:** Data for training, testing, and validation.

Class	Training	Validation	Testing	Total
COVID-19	340	37	111	488
CAP	340	49	111	500
Normal	340	49	111	500

**Table 7:** Confusion matrix and classification report for different machine learning classifiers.

Confusion matrix						Classification report				
Models	Category	COVID-19	CAP	Normal	Total	Category	Precision	Recall	F1Score	Specificity
SVM	COVID-19	104	7	0	111	COVID-19	0.93	0.92	0.92	0.96
	CAP	7	103	2	111	CAP	0.92	0.88	0.89	0.96
	Normal	1	6	104	111	Normal	0.93	0.98	0.95	0.96
	Total	112	116	106	333	Average	0.92	0.93	0.92	0.96

Random Forest	COVID-19	106	5	0	111	COVID-19	0.95	0.97	0.95	0.97
	CAP	3	106	2	111	CAP	0.95	0.95	0.95	0.97
	Normal	0	6	105	111	Normal	0.95	0.98	0.95	0.97
	**Total**	**109**	**111**	**107**	**333**	**Average**	**0.95**	**0.96**	**0.95**	**0.97**

Decision Tree	COVID-19	104	5	2	111	COVID-19	0.93	0.93	0.93	0.96
	CAP	3	103	5	111	CAP	0.92	0.91	0.91	0.96
	Normal	4	4	103	111	Normal	0.92	0.93	0.92	0.96
	Total	111	112	110	333	Average	0.92	0.92	0.92	0.96

Naive Bayes	COVID-19	82	20	9	111	COVID-19	0.73	0.86	0.79	0.87
	CAP	8	83	20	111	CAP	0.74	0.62	0.67	0.86
	Normal	5	29	77	111	Normal	0.69	0.72	0.70	0.87
	Total	95	132	106	333	Average	0.72	0.73	0.72	0.87

KNN	COVID-19	104	7	0	111	COVID-19	0.93	0.91	0.91	0.97
	CAP	6	103	2	111	CAP	0.92	0.88	0.89	0.97
	Normal	4	6	101	111	Normal	0.90	0.98	0.93	0.96
	Total	114	117	103	333	Average	0.92	0.92	0.92	0.97

**Table 8:** Confusion matrix and classification report for various deep learning classifiers.

Confusion matrix						Classification report				
Models	Category	COVID-19	CAP	Normal	Total	Category	Precision	Recall	F1Score	Specificity
CNN	COVID-19	100	5	6	111	COVID-19	0.90	0.90	0.90	0.95
	CAP	5	101	4	111	CAP	0.91	0.89	0.89	0.95
	Normal	5	7	99	111	Normal	0.89	0.90	0.89	0.95
	Total	111	113	109	333	Average	0.90	0.90	0.90	0.95

AlexNet	COVID-19	105	3	3	111	COVID-19	0.94	0.94	0.94	0.97
	CAP	2	106	3	111	CAP	0.95	0.95	0.95	0.97
	Normal	4	2	104	111	Normal	0.93	0.93	0.93	0.97
	**Total**	**111**	**111**	**111**	**333**	**Average**	**0.94**	**0.94**	**0.94**	**0.97**

VGG-16	COVID-19	104	4	3	111	COVID-19	0.93	0.93	0.93	0.96
	CAP	3	104	4	111	CAP	0.93	0.93	0.93	0.95
	Normal	4	3	104	111	Normal	0.93	0.93	0.93	0.96
	Total	111	111	111	333	Average	0.93	0.93	0.93	0.96

Resnet50	COVID-19	102	6	3	111	COVID-19	0.92	0.93	0.92	0.96
	CAP	5	101	5	111	CAP	0.91	0.90	0.90	0.95
	Normal	3	6	102	111	Normal	0.92	0.93	0.92	0.96
	Total	110	113	110	333	Average	0.92	0.92	0.92	0.96

Inception v3	COVID-19	100	4	7	111	COVID-19	0.90	0.89	0.89	0.94
	CAP	6	100	5	111	CAP	0.90	0.91	0.90	0.95
	Normal	6	6	99	111	Normal	0.89	0.89	0.89	0.95
	Total	112	110	111	333	Average	0.90	0.90	0.90	0.95

**Table 9:** Confusion matrix and classification report for the proposed work, CNN for feature extraction, and various machine learning models for classification.

Confusion matrix						Classification report				
Models	Category	COVID-19	CAP	Normal	Total	Category	Precision	Recall	F1Score	Specificity
CNN+SVM	COVID-19	104	4	3	111	COVID-19	0.93	0.93	0.93	0.97
	CAP	3	104	4	111	CAP	0.93	0.93	0.93	0.97
	Normal	4	3	104	111	Normal	0.93	0.93	0.93	0.97
	Total	111	113	109	333	Average	0.93	0.93	0.93	0.97

CNN+Random Forest	COVID-19	104	3	4	111	COVID-19	0.94	0.93	0.93	0.96
	CAP	3	103	5	111	CAP	0.93	0.93	0.93	0.96
	Normal	5	5	101	111	Normal	0.91	0.92	0.91	0.97
	Total	112`	111	110	333	Average	0.93	0.93	0.93	0.96

CNN+Decision Tree	COVID-19	101	4	6	111	COVID-19	0.91	0.92	0.92	0.95
	CAP	3	101	7	111	CAP	0.91	0.92	0.92	0.96
	Normal	5	5	101	111	Normal	0.91	0.89	0.90	0.96
	Total	109	110	114	333	Average	0.91	0.91	0.91	0.96

CNN+Naive Bayes	COVID-19	104	3	4	111	COVID-19	0.94	0.93	0.93	0.91
	CAP	3	103	5	111	CAP	0.93	0.93	0.93	0.92
	Normal	5	5	101	111	Normal	0.91	0.92	0.91	0.91
	Total	112	111	110	333	Average	0.93	0.93	0.93	0.91

CNN+KNN	COVID-19	102	5	4	111	COVID-19	0.92	0.91	0.91	0.95
	CAP	4	102	5	111	CAP	0.91	0.91	0.91	0.95
	Normal	5	4	102	111	Normal	0.91	0.91	0.91	0.96
	Total	111	111	110	333	Average	0.91	0.91	0.91	0.95

**Table 10:** Confusion matrix and Classification report for the proposed work, AlexNet for feature extraction, and various machine learning models for classification.

Confusion matrix						Classification report				
Models	Category	COVID-19	CAP	Normal	Total	Category	Precision	Recall	F1Score	Specificity
AlexNet+SVM	COVID-19	107	4	0	111	COVID-19	0.96	0.96	0.96	0.98
	CAP	1	108	2	111	CAP	0.95	0.97	0.96	0.97
	Normal	1	3	107	111	Normal	0.98	0.96	0.97	0.98
	**Total**	**109**	**113**	**109**	**333**	**Average**	**0.96**	**0.96**	**0.96**	**0.98**

AlexNet+Random Forest	COVID-19	102	5	4	111	COVID-19	0.92	0.92	0.91	0.96
	CAP	4	102	5	111	CAP	0.91	0.91	0.91	0.98
	Normal	5	4	102	111	Normal	0.91	0.91	0.91	0.98
	Total	111	111	110	333	Average	0.91	0.91	0.91	0.98

AlexNet+Decision Tree	COVID-19	104	4	3	111	COVID-19	0.94	0.94	0.94	0.97
	CAP	3	103	4	111	CAP	0.93	0.93	0.93	0.97
	Normal	4	3	104	111	Normal	0.93	0.93	0.93	0.96
	Total	111	111	111	333	Average	0.94	0.94	0.94	0.97

AlexNet+Naive Bayes	COVID-19	93	8	10	111	COVID-19	0.84	0.82	0.83	0.91
	CAP	10	93	8	111	CAP	0.84	0.83	0.83	0.90
	Normal	10	11	90	111	Normal	0.81	0.83	0.82	0.91
	Total	113	112	108	333	Average	0.83	0.83	0.83	0.91

AlexNet + KNN	COVID-19	104	3	4	111	COVID-19	0.94	0.94	0.94	0.94
	CAP	4	103	40	111	CAP	0.93	0.94	0.93	0.93
	Normal	3	4	104	111	Normal	0.94	0.93	0.93	0.94
	Total	111	110	112	333	Average	0.94	0.94	0.94	0.94

**Table 11:** Confusion matrix and classification report for proposed work, VGG-16 for feature extraction, and different machine learning models for classification.

Confusion matrix						Classification report				
Models	Category	COVID-19	CAP	Normal	Total	Category	Precision	Recall	F1Score	Specificity
VGG-16+SVM	COVID-19	105	2	4	111	COVID-19	0.95	0.95	0.95	0.97
	CAP	3	105	3	111	CAP	0.94	0.95	0.94	0.97
	Normal	2	4	105	111	Normal	0.94	0.95	0.94	0.97
	Total	110	111	112	333	Average	0.94	0.94	0.94	0.97

VGG-16+Random Forest	COVID-19	106	2	3	111	COVID-19	0.95	0.95	0.96	0.97
	CAP	3	106	2	111	CAP	0.95	0.95	0.95	0.97
	Normal	3	3	105	111	Normal	0.94	0.95	0.94	0.97
	Total	112	111	110	333	Average	0.95	0.95	0.95	0.97

VGG-16+Decision Tree	COVID-19	104	4	3	111	COVID-19	0.94	0.94	0.94	0.96
	CAP	3	103	4	111	CAP	0.93	0.94	0.93	0.96
	Normal	3	4	104	111	Normal	0.94	0.93	0.93	0.96
	Total	111	110	112	333	Average	0.94	0.94	0.93	0.96

VGG-16+Naive Bayes	COVID-19	94	7	10	111	COVID-19	0.85	0.86	0.86	0.93
	CAP	7	94	10	111	CAP	0.85	0.85	0.85	0.92
	Normal	8	9	94	111	Normal	0.85	0.82	0.84	0.93
	Total	109	110	114	333	Average	0.85	0.84	0.85	0.92

VGG-16+KNN	COVID-19	103	4	4	111	COVID-19	0.93	0.94	0.93	0.96
	CAP	3	103	4111	333	CAP	0.94	0.93	0.93	0.96
	Normal	4	4	103	111	Normal	0.93	0.94	0.93	0.96
	Total	110	112	110	333	Average	0.93	0.94	0.93	0.96

**Table 12:** Confusion matrix and classification report for proposed work, Resnet50 for feature extraction, and machine learning models for classification.

Confusion matrix						Classification report				
Models	Category	COVID-19	CAP	Normal	Total	Category	Precision	Recall	F1Score	Specificity
Resnet50+SVM	COVID-19	104	4	3	111	COVID-19	0.94	0.95	0.94	0.94
	CAP	3	104	4	111	CAP	0.94	0.93	0.93	0.93
	Normal	3	4	104	111	Normal	0.94	0.94	0.94	0.94
	Total	110	112	111	333	Average	0.94	0.94	0.94	0.94

Resnet50+Random Forest	COVID-19	105	3	3	111	COVID-19	0.95	0.95	0.95	0.97
	CAP	3	104	4	111	CAP	0.94	0.95	0.95	0.97
	Normal	3	3	105	111	Normal	0.94	0.94	0.95	0.97
	**Total**	**111**	**110**	**111**	**333**	**Average**	**0.95**	**0.95**	**0.95**	**0.97**

Resnet50+Decision Tree	COVID-19	102	4	5	111	COVID-19	0.92	0.93	0.93	0.96
	CAP	4	103	4	111	CAP	0.93	0.92	0.93	0.96
	Normal	4	5	102	111	Normal	0.92	0.92	0.92	0.96
	Total	110	11	111	333	Average	0.92	0.92	0.92	0.96

Resnet50+Naive Bayes	COVID-19	97	7	7	111	COVID-19	0.87	0.87	0.87	0.94
	CAP	7	96	8	111	CAP	0.88	0.86	0.87	0.94
	Normal	8	6	97	111	Normal	0.86	0.86	0.86	0.93
	Total	112	109	112	333	Average	0.87	0.86	0.86	0.94

Resnet50+KNN	COVID-19	102	5	4	111	COVID-19	0.92	0.92	0.92	0.95
	CAP	5	101	5	111	CAP	0.91	0.92	0.91	0.96
	Normal	4	4	103	111	Normal	0.93	0.92	0.93	0.96
	Total	111	110	112	333	Average	0.92	0.92	0.92	0.96

**Table 13:** Confusion matrix and classification report for proposed work, Inception V3 for feature extraction, and various machine learning models for classification.

Confusion matrix						Classification report				
Models	Category	COVID-19	CAP	Normal	Total	Category	Precision	Recall	F1Score	Specificity
IncpetionV3+SVM	COVID-19	103	4	4	111	COVID-19	0.93	0.93	0.93	0.95
	CAP	4	102	5	111	CAP	0.92	0.93	0.92	0.96
	Normal	4	4	103	111	Normal	0.93	0.92	0.94	0.96
	Total	111	110	11	333	Average	0.93	0.93	0.93	0.96

Inception V3 + Random Forest	COVID-19	102	5	4	111	COVID-19	0.92	0.92	0.92	0.96
	CAP	4	103	4	111	CAP	0.91	0.91	0.91	0.96
	Normal	5	5	101	111	Normal	0.91	0.92	0.91	0.95
	Total	111	113	109	333	Average	0.91	0.92	0.92	0.96

InceptionV3+Decision Tree	COVID-19	101	5	5	111	COVID-19	0.91	0.91	0.91	0.95
	CAP	5	101	5	111	CAP	0.91	0.90	0.91	0.95
	Normal	5	6	100	111	Normal	0.90	0.90	0.90	0.95
	Total	111	112	110	333	Average	0.91	0.90	0.91	0.95

InceptionV3+Naive Bayes	COVID-19	96	9	6	111	COVID-19	0.86	0.86	0.86	0.93
	CAP	6	96	9	111	CAP	0.86	0.85	0.86	0.93
	Normal	7	9	95	111	Normal	0.89	0.88	0.88	0.93
	Total	108	112	107	333	Average	0.87	0.87	0.87	0.93

InceptionV3+KNN	COVID-19	101	5	5	111	COVID-19	0.91	0.92	0.91	0.95
	CAP	4	102	5	111	CAP	0.92	0.91	0.92	0.95
	Normal	5	5	101	111	Normal	0.96	0.91	0.91	0.95
	Total	110	112	111	333	Average	0.91	0.92	0.92	0.95

**Table 14:** Performance analysis with and without image preprocessing.

Model	With preprocessing					Without preprocessing				
	Accuracy	F1-score	MAE	RMSE	Specificity	Accuracy	F1-score	MAE	RMSE+	Specificity
SVM	93.39%	0.92	0.229	0.054	0.96	91.11%	0.91	0.298	0.089	0.94
Random Forest	95.19%	0.95	0.218	0.049	0.97	94.23%	0.94	0.227	0.054	0.96
Decision Tree	93.12%	0.92	0.262	0.063	0.96	92.02%	0.92	0.265	0.077	0.94
Naive Bayes	72.69%	0.72	0.795	0.396	0.87	69.63%	0.69	0.803	0.412	0.78
KNN	92.49%	0.92	0.226	0.051	0.97	90.15%	0.90	0.321	0.093	0.92
CNN	90.01%	0.90	0.314	0.087	0.95	89.45%	0.89	0.356	0.097	0.92
AlexNet	94.59%	0.94	0.206	0.061	0.97	93.78%	0.93	0.226	0.049	0.94
VGG-16	93.69%	0.93	0.227	0.051	0.96	91.82%	0.92	0.225	0.051	0.93
Resnet50	91.59%	0.91	0.272	0.077	0.96	91.18%	0.91	0.299	0.065	0.93
InceptionV3	89.78%	0.89	0.313	0.082	0.95	87.43%	0.86	0.391	0.099	0.94
CNN+SVM	91.12%	0.91	0.281	0.082	0.97	88.73%	0.89	0.366	0.096	0.93
CNN+Random Forest	92.49%	0.93	0.227	0.052	0.97	89.99%	0.90	0.325	0.088	0.93
CNN+Decision Tree	90.99%	0.91	0.271	0.078	0.96	88.31%	0.88	0.347	0.093	0.93
CNN+Naive Bayes	82.85%	0.83	0.456	0.123	0.91	79.56%	0.80	0.478	0.178	0.87
CNN+KNN	91.89%	0.92	0.253	0.061	0.95	89.56%	0.89	0.312	0.079	0.90
AlexNet+SVM	**96.69%**	**0.97**	**0.217**	**0.043**	**0.98**	**95.12%**	**0.95**	**0.217**	**0.047**	**0.93**
AlexNet+Random Forest	96.09%	0.96	0.225	0.049	0.98	95.11%	0.95	0.213	0.047	0.95
AlexNet+Decision Tree	93.09%	0.93	0.225	0.050	0.97	92.45%	0.92	0.223	0.053	0.92
AlexNet+Naive Byes	83.13%	0.83	0.421	0.099	0.91	80.55%	0.81	0.492	0.153	0.86
AlexNet+KNN	93.39%	0.93	0.220	0.055	0.94	90.91%	0.91	0.279	0.050	0.91
VGG-16+SVM	94.59%	0.95	0.205	0.061	0.97	93.69%	0.93	0.221	0.045	0.93
VGG-16+Random Forest	95.19%	0.95	0.200	0.054	0.97	93.34%	0.93	0.22	0.049	0.94
VGG-16+Decision Tree	93.39%	0.93	0.214	0.043	0.96	91.23%	0.91	0.277	0.080	0.93
VGG-16+Naive Bayes	84.68%	0.85	0.419	0.083	0.92	82.87%	0.83	0.455	0.122	0.88
VGG-16+KNN	93.09%	0.93	0.261	0.062	0.96	92.45%	0.92	0.227	0.053	0.92
Resnet50+SVM	93.69%	0.94	0.227	0.050	0.97	91.78%	0.93	0.220	0.048	0.94
Resnet50+Random Forest	94.29%	0.94	0.201	0.059	0.97	86.45%	0.86	0.399	0.087	0.93
Resnet50+Decision Tree	92.19%	0.91	0.278	0.079	0.96	89.10%	0.89	0.369	0.101	0.93
Resnet50+Naive Bayes	87.08%	0.87	0.389	0.100	0.94	85.18%	0.85	0.402	0.077	0.90
Resnet50+KNN	91.89%	0.92	0.249	0.058	0.96	88.99%	0.89	0.337	0.088	0.91
InceptionV3+SVM	92.79%	0.93	0.220	0.047	0.99	89.99%	0.90	0.319	0.091	0.92
InceptionV3+Random Forest	91.89%	0.92	0.236	0.059	0.96	87.91%	0.88	0.320	0.079	0.93
InceptionV3+Decision Tree	90.69%	0.91	0.266	0.070	0.95	88.45%	0.88	0.340	0.091	0.91
InceptionV3+Naive Bayes	86.18%	0.86	0.411	0.078	0.93	84.72%	0.85	0.416	0.081	0.91
InceptionV3+KNN	91.29%	0.91	0.288	0.075	0.95	90.11%	0.90	0.311	0.090	0.93

## Data Availability

MedRvix BioRvix NEJM JAMA Lancet https://www.kaggle.com/c/covidct/data Radiopedia https://radiopaedia.org/articles/imaging-data-sets-artificial-intelligence Zenodo https://zenodo.org/record/3757476#.YBVbtWRN0zQ GitHub https://github.com/ieee8023/covid-chestxray-dataset.

## References

[1] Zhu N., Zhang D., Wang W. (2020). A novel coronavirus from patients with pneumonia in China, 2019. *New England Journal of Medicine*.

[2] WHO (2020). *Coronavirus Disease 2019 (COVID-19) Situation Report–39*.

[3] Shi H., Xiaoyu Han N., Jiang Y., Cao O., Alwalid J., Zheng C. (2020). Radiological findings from 81 patients with COVID-19 pneumonia in Wuhan, China: a descriptive study. *The Lancet Infectious Diseases*.

[4] Pan F., Ye T., Sun P. (2020). Time course of lung changes at chest ct during recovery from coronavirus disease 2019 (COVID-19). *Radiology*.

[5] Xie X., Zhong Z., Zhao W., Zheng C., Wang F., Liu J. (2020). Chest ct for typical coronavirus disease 2019 (COVID-19) pneumonia: relationship to negative rt-pcr testing. *Radiology*.

[6] Huang P., Liu T., Huang L. (2020). Use of chest CT in combination with negative RT-PCR assay for the 2019 novel coronavirus but high clinical suspicion. *Radiology*.

[7] Fang Y., Zhang H., Xie J. (2020). Sensitivity of chest CT for COVID-19: com-parison to RT-PCR. *Radiology*.

[8] Sun Q., Xu X., Xie J., Li J., Huang X. (2020). Evolution of computed tomography manifestations in five patients who recovered from coronavirus disease 2019 (COVID-19) pneumonia. *Korean Journal of Radiology*.

[9] Ai T., Yang Z., Hou H. (2020). Correlation of chest ct and RT-PCR testing for coronavirus disease 2019 (COVID-19) in China: a report of 1014 cases. *Radiology*.

[10] Committee, NH: General office of national health committee (2020). *Notice on the Issuance of a Program for the Diagnosis and Treatment of Novel Coronavirus (2019-ncov) Infected Pneumonia (Trial Revised Fifth Edition)*.

[11] Xiaowei X., Xiangao J., Chunlian M. (2020). Deep learning system to screen coronavirus disease 2019 pneumonia.

[12] Zhao J., He X., Yang X., Zhang Y., Zhang S., Xie P. (2020). Covid-ct-dataset: a ct scan dataset about covid-19. https://arxiv.org/abs/2003.13865.

[13] Shah V., Keniya R., Shridharani A., Punjabi M., Shah J., Mehendale N. (2021). Diagnosis of COVID-19 using CT scan images and deep learning techniques. *Emergency Radiology*.

[14] He X., Yang X., Zhang S. (2020). *Sample-Efficient Deep Learning for COVID-19 Diagnosis Based on CT Scans*.

[15] Horry M. J., Chakraborty S., Paul M. (2020). COVID-19 detection through transfer learning using multimodal imaging data. *IEEE Access*.

[16] Song Y., Zheng S., Li L. (2020). *Deep Learning Enables Accurate Diagnosis of Novel Coronavirus (COVID-19) with CT Images*.

[17] Jianpeng Z., Yutong X., Yi L., Chunhua S., Yong X. (2020). Covid-19 screening on chest x-ray images using deep learning based anomaly detection. https://arxiv.org/abs/2003.12338.

[18] Ali N., Ceren K., Ziynet P. (2020). Automatic detection of coronavirus disease (covid-19) using x-ray images and deep convolutional neural networks. https://arxiv.org/abs/2003.10849.

[19] Hemdan E. E. D., Shouman M. A., Karar M. E. (2020). Covidx-net: a framework of deep learning classifiers to diagnose covid-19 in x-ray images. https://arxiv.org/abs/2003.11055.

[20] Apostolopoulos I. D., Mpesiana T. A. (2020). Covid-19: automatic detection from x-ray images utilizing transfer learning with convolutional neural networks. *Physical and Engineering Sciences in Medicine*.

[21] Sethy P., Behera S. (2020). *Detection of Coronavirus Disease (COVID-19) Based on Deep Features*.

[22] Abbas A., Abdelsamea M. M., Gaber M. M. (2020). Classification of COVID-19 in chest X-ray images using DeTrac deep convolutional neural network. https://arxiv.org/abs/2003.13815.

[23] Afshar P., Heidarian S., Naderkhani F., Oikonomou A., Plataniotis K. N., Mohammadi A. (2020). Covid-caps: a capsule network-based framework for identification of covid-19 cases from x-ray images. https://arxiv.org/abs/2004.02696.

[24] Hall L. O., Paul R., Goldgof D. B., Goldgof G. M. (2020). Finding covid-19 from chest x-rays using deep learning on a small dataset. https://arxiv.org/abs/2004.02060.

[25] Farooq M., Hafeez A. (2020). Covid-resnet: a deep learningframework for screening of covid 19 from radiographs. https://arxiv.org/abs/2003.14395.

[26] Maghdid H. S., Ghafoor K. Z., Sadiq A. S., Cur-ran K., Rabie K. (2020). A novel AI-enabled framework to diagnose coronavirus COVID-19 using smartphone embedded sensors: design study. https://arxiv.org/abs/2003.07434.

